# Short-term and long-term safety and efficacy of tenofovir alafenamide, tenofovir disoproxil fumarate and entecavir treatment of acute-on-chronic liver failure associated with hepatitis B

**DOI:** 10.1186/s12879-021-06237-x

**Published:** 2021-06-14

**Authors:** Juan Li, Chunhua Hu, Yi Chen, Rou Zhang, Shan Fu, Mimi Zhou, Zhijie Gao, Mengjun Fu, Taotao Yan, Yuan Yang, Jianzhou Li, Jinfeng Liu, Tianyan Chen, Yingren Zhao, Yingli He

**Affiliations:** 1 Department of Infectious Diseases, First Affiliated Teaching Hospital, School of Medicine, Xi’an Jiaotong University, Yanta Road (w), No. 277, Xi’an City, 710061 Shaanxi Province China; 2 Institution of Hepatology, First Affiliated Teaching Hospital, School of Medicine, Xi’an Jiaotong University, Xi’an, 710061 Shaanxi province China; 3 Shaanxi Clinical Research Center of Infectious Diseases, Xi’an, 710061 Shaanxi province China

**Keywords:** Tenofovir alafenamide, Tenofovir disoproxil fumarate, Entecavir, Hepatitis B virus, Acute-on-chronic liver failure

## Abstract

**Background & Aims:**

There is limited evidence on the efficacy and safety of nucleos(t) ide analogues (NAs) in the treatment of HBV-ACLF. Our objective was to evaluate the outcomes among TAF, TDF and ETV, three first-line antivirals against chronic hepatitis B, in patients with HBV-ACLF.

**Methods:**

Patients with HBV-related ACLF were recruited and received daily TAF (25 mg/d), TDF (300 mg/d) and ETV (0.5 mg/d). They were prospectively followed-up. The primary endpoint was overall survival at week 12 and week 48, the secondary endpoints were virological response and biochemical response.

**Results:**

Forty gender and age matched eligible subjects were recruited and divided into three groups: TAF group, TDF group and ETV group. By week 48, 8 (80%) patients in TAF group, 6 (60%) patients in TDF group and 17 (85%) patients in ETV group survived without liver transplantation (*P* = 0.251). After 4 weeks of NAs treatment, all three groups showed paralleling reduction of HBV DNA levels. All three groups presented similar biochemical responses at week 4, patients treated with TAF showed a priority in total bilirubin reduction, albumin and cholesterol maintenance. Additionally, although there was no significant difference in changes of serum urea, serum creatinine, serum cystatin C and estimated GFR among the three groups by treatment week 4, TDF showed unfavorable renal safety even in short -term treatment. The treatment using NAs was well-tolerated and there was no serious drug-related adverse event reported.

**Conclusions:**

TAF, TDF and ETV are of similar efficacy and safety in short-term and long-term treatment of HBV-ACLF.

**Trial registration:**

This study is ongoing and is registered with ClinicalTrials.gov, NCT03640728 (05/02/2019).

## Introduction

HBV-related acute-on-chronic liver failure (ACLF) is a severe clinical syndrome remaining an extremely high mortality rate with limited effective medical intervention [1–3]. Although liver transplantation is a life-saving option for ACLF, the obstruction in finding a matching donor and the high cost hinder its extensive clinical use.

HBV replication is one of the key risk factors leading to progression from liver damage to liver failure [4–6]. There were limited studies indicating the efficacy and safety of nucleos(t) ide analogues on HBV-associated ACLF patients. Potent antivirals like entecavir (ETV), tenofovir disoproxil fumarate (TDF) and tenofovir alafenamide (TAF) are now recommended as first-line therapy for patients with chronic HBV infection based on their significant suppression of viral replication and high barriers to virus resistance [7–9]. Although current clinical guidelines recommend early intervention using oral antiviral treatment in HBV-related ACLF, [2, 7, 8] the specific strategy of antiviral treatment is still unclear in newly approved TAF and some details of TDF in hepatorenal syndrome conditions, therefore data gap still exists with little evidence.

Despite the inconsistent results, numerous studies have reported the efficacy of ETV on the survival of HBV-ACLF patients. Compared to controls without antivirals, studies declared no difference in short-term survival after 3-month of ETV treatment, [10, 11] while others reported improved survival [12, 13]. When compared with lamivudine (LAM), studies showed similar survival rates after 3 months ETV treatment, [14, 15] while *Wong* et al. [16] indicated that entecavir treatment was independently associated with increased short-term mortality. Notably, a Germany study reported that ETV treated 16 patients with decompensated liver cirrhosis or liver failure, 5 of them had lactic acidosis, which cannot be ruled out as a side effect of the drug [17].

Unlike ETV, data are limited on the efficacy of TDF treatment in HBV-related ACLF. *Garg* et al. [18] demonstrated that TDF significantly improved the 3-month outcomes of patients with HBV-related ACLF as compared with placebo. *Wan* et al. [19] demonstrated that TDF was superior to ETV in treating HBV-ACLF via rapid viral suppression, improving liver function and short-term survival. However, the excessive circulating tenofovir produced following oral intake of TDF was reported to cause renal and bone toxicity over long-term use, which cannot be ignored especially in aging population. Notably, patients with ACLF are likely to combine hepatorenal syndrome and even be at risk of renal failure.

Tenofovir alafenamide is a novel oral phosphonamidase prodrug of tenofovir, providing liver-targeted high intracellular concentrations of tenofovir diphosphate while reduced systematic exposure of tenofovir [20]. Thus, the oral dose can be lowered substantially from 300 mg TDF to 25 mg TAF daily. In two double-blinded phase 3 pivotal trials, [21, 22] the noninferiority of TAF vs. TDF in terms of virologic efficacy has been demonstrated. Pharmacodynamic studies have suggested that lower circulating tenofovir levels also reduce drug load in the kidneys and the bones, which improves renal and bone safety [20]. Therefore, TAF is expected to provide the similar clinical efficacy while improve tolerability and safety. To the best of our knowledge, there is so far no clinical study evaluating the outcomes of TAF treatment in patients with HBV-related ACLF from literatures.

In the present study, we aim to prospectively investigate the safety and efficacy of TAF, TDF and ETV in patients with HBV-ACLF.

## Materials and methods

### Study design and participants

From January 2019 to December 2019, consecutive HBV-related ACLF patients treated with TAF, TDF or ETV monotherapy in the First Affiliated Teaching Hospital of Xi’an Jiaotong University, the biggest general hospital in northwest China under the direct administration of the Chinese Ministry of Health, were recruited in this study. The inclusion criteria were: (1) age 18–70 years; Hepatitis B surface antigen positive≥6 months; (2) ACLF was diagnosed according to the diagnostic criteria recommended by the Asian Pacific Association for the Study of the Liver (APASL) [2]. Patients were excluded if they had any of the following conditions: (1) concomitant hepatitis A, C, D, E virus, or other hepadnaviruses infections; (2) malignancies, such as hepatocellular carcinoma; (3) with one or more additional known primary or secondary causes of liver disease, other than hepatitis B.

During hospitalization, antiviral therapy with TAF, TDF or ETV was started immediately when HBV-DNA was tested positive. Adverse side effects were carefully monitored during the study period. All patients were given standard medical treatment, including absolute bed rest, supportive care, energy supplements and vitamins. Therapeutic plasma exchange (PE) or double plasma molecular absorption system (DPMAS) were administered for patients at doctors’ discreet decision.

This prospective cohort study was conducted in accordance with the Declaration of Helsinki and the protocol was approved by the Ethics Committee of the First Affiliated Teaching Hospital of Xi’an Jiaotong University.

### Follow-up and laboratory examinations

During the hospital stay, patients were closely monitored, including clinical assessments, complete blood count, liver function tests (alanine aminotransferase, total bilirubin, serum albumin), renal function (serum urea, creatinine, eGFR), electrolytes (serum sodium, potassium) and coagulation (international normalized ratio), HBV DNA load (COBAS TaqMan, lower detection limit 20 IU/mL). The tests were performed at the central lab of the hospital. Liver disease severity was assessed daily using the Model for End-Stage Liver Disease (MELD) score [23] and Child-Turcotte-Pugh score (CTP) [24]. The follow-up visits were scheduled at the 12, 24 and 48 weeks, or whenever the patients felt unwell.

### Endpoints

The primary endpoints were liver transplant-free survival at week 12 and week 48. The secondary endpoints were virologic response, biochemical response, and adverse events during the study.

### Statistical analysis

Statistical analysis was performed using SPSS 23.0 for Windows (SPSS, Chicago, IL), with graphs drawn using GraphPad Prism 8.0 (GraphPad, La Jolla, Calif). Quantitative data were expressed as mean ± standard deviation (SD), interquartile range (IQR) or median (range), and the categorical data were expressed as the number (percentage). One-way analysis of variance (ANOVA), t-test, or the nonparametric Mann–Whitney U test was used where appropriate. A Pearson’s Chi-square or Fisher’s exact test was performed for comparison of qualitative data. Actuarial probabilities of death or liver transplantation during follow-up were calculated by Kaplan-Meier method and compared by log-rank test. Results with a two-tailed *p* value of < 0.05 considered statistically significant.

## Results

### Demographic and clinical baseline characteristics of the study population

**Fig. 1:**
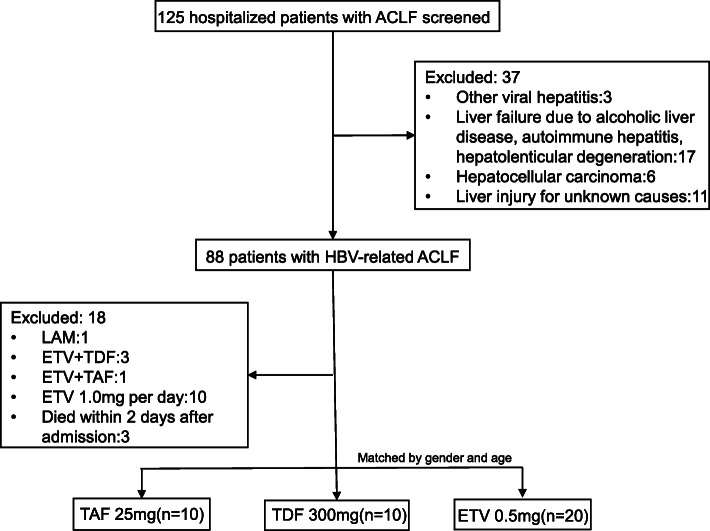
Patient disposition during the study. ACLF, acute-on-chronic liver failure; LAM, lamivudine; ETV, entecavir; TDF, tenofovir disoproxil fumarate; TAF, tenofovir alafenamide

We screened 125 ACLF patients during this period, and 88 HBV-related ACLF people were identified. The following patients were excluded:1 patient was treated with lamivudine, 4 patients with combination treatment of two antiviral agents, 10 received 1.0 mg entecavir daily and 3 died within 2 days after admission. Finally, gender and age matched 10 patients with TAF, 10 with TDF and 20 with ETV treatment were enrolled (Fig. 1). They were prospectively followed-up regularly till death or end of the study.

The primary baseline demographics and disease characteristics were summarized in Table 1. There were no significant differences among three groups in the baseline characteristics of age, gender, serum ALT, AST, WBC, PLT, albumin, urea, creatinine, sodium, eGFR, HBV DNA viral load, CTP score, MELD score, and complications.

**Table 1:** Baseline characteristics of the study population

Parameters	TAF 25 mg	TDF 300 mg	ETV 0.5 mg	*P* value
	(*n* = 10)	(*n* = 10)	(*n* = 20)	
Age (years)	40.56 ± 11.18	41.00 ± 12.64	39.72 ± 9.13	0.901
Male, *n* (%)	10 (100)	10 (100)	20 (100)	1.0
Liver cirrhosis	8 (80)	7 (70)	13 (65)	0.901
ALT (U/L)	351.10 (308.72)	203.67 (173.61)	385.85 (519.33)	0.588
AST (U/L)	263.78 (242.47)	156.89 (84.39)	382.61 (397.06)	0.707
TBIL (μmol/L)	362.93 (178.62)	321.10 (127.87)	240.44 (144.35)	0.174
Albumin (g/L)	31.32 (3.71)	30.90 (4.38)	32.23 (4.19)	0.905
Urea (mmol/L)	5.71 (3.09)	5.74 (2.70)	5.86 (3.23)	0.948
Creatinine (μmol/L)	77.22 (34.20)	66.22 (22.08)	66.00 (24.27)	0.458
eGFR (mL/min/1.73m^2^)	112.76 (41.47)	150.23 (30.12)	112.34 (68.85)	0.274
INR	1.71 (0.32)	2.08 (0.87)	2.13 (0.63)	0.179
WBC (× 10^9^/L)	6.22 (1.82)	7.28 (3.48)	6.91 (3.54)	0.819
PLT (×10^9^/L)	115.11 (60.48)	97.44 (113.02)	98.33 (63.09)	0.168
Serum sodium, mmol/L	137.11 (2.32)	135.11 (4.96)	135.16 (7.06)	0.784
HbeAg positive	8 (80)	8 (80)	15 (75)	0.931
LogHBV DNA (log IU/mL)	4.80 (1.93)	5.81 (1.87)	5.13 (1.89)	0.458
MELD score	21.56 (5.66)	21.33 (7.28)	20.50 (5.40)	0.943
CTP score	10.22 (1.44)	10.78 (1.64)	10.44 (1.56)	0.151
CTP class(B/C)	3/7	1/9	1/19	0.202
NUC-naïve	7 (70)	2 (20)	15 (75)	0.019
Follow-up (wk)	68.50 (48–92)	64.50 (51–95)	69.00 (50–95)	0.938

Data are expressed as mean ± standard deviation (SD), number (percentage) or median (range)

*ETV* entecavir, *TDF* tenofovir disoproxil fumarate, *TAF* tenofovir alafenamide, *ALT* alanine aminotransferase, *AST* aspartate Aminotransferase, *TBIL* total bilirubin, *INR* international normalized ratio, *WBC* white blood cell count, *PLT* platelet count, *HBeAg* Hepatitis B e antigen, *HBV* hepatitis B virus, *MELD* Model for End-stage Liver Disease, *CTP* Child-Turcotte-Pugh, *NUC* nucleos(t) ide analogues

### Overall mortality or liver transplantation and liver-related complications

**Fig. 2:**
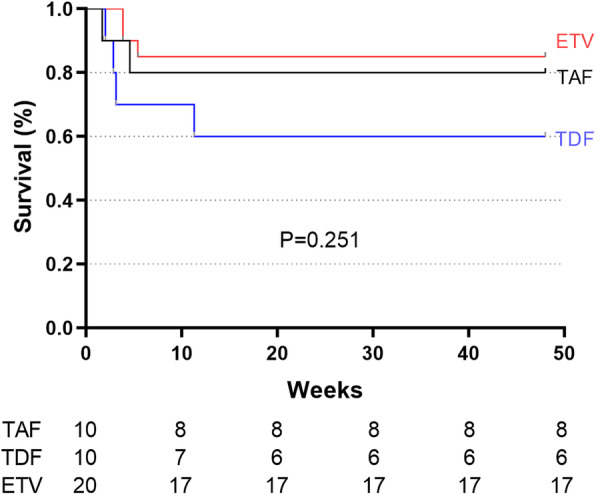
Cumulative incidences of mortality during treatment with tenofovir alafenamide, tenofovir disoproxil fumarate and entecavir in patients with HBV-related ACLF. ETV, entecavir; TAF, tenofovir alafenamide; TDF, tenofovir disoproxil fumarate

By week 12, 2 (20%) patients in TAF group, 4 (40%) patients in TDF group and 3 (15%) patients in ETV group died or underwent liver transplantation (Table 2). Of these, 66.7% (*n* = 6) of the deaths or liver transplantations occurred within 28 days (Table 2). As shown in Table 2, the patients in three groups had comparable rates of liver-related complications, including ascites, spontaneous bacterial peritonitis, infection, gastrointestinal hemorrhage, hepatic encephalopathy and hepatorenal syndrome. Median follow-up period was 67 weeks. The cumulative rates of overall mortality or liver transplantation were similar among the TAF, TDF and ETV groups by week 48. (*P* = 0.251, Fig. 2).

**Table 2:** Clinical outcomes of patients with HBV-related acute-on-chronic liver failure on tenofovir alafenamide, tenofovir disoproxil fumarate and entecavir treatment

Outcome, *n* (%)	TAF 25 mg	TDF 300 mg	ETV 0.5 mg	*P* value
	(*n* = 10)	(*n* = 10)	(*n* = 20)	
Mortality or transplantation				
Within 28 days	1 (10)	3 (30)	2 (10)	0.504
Within 3 months	2 (20)	4 (40)	3 (15)	0.320
Within 48 weeks	2 (20)	4 (40)	3 (15)	0.320
Liver-related complications (first 3 months)				
Ascites	7 (70)	7 (70)	13 (65)	0.945
Spontaneous bacterial peritonitis	6 (60)	6 (60)	11 (55)	0.950
Infection during treatment	3 (30)	4 (40)	7 (35)	0.896
Gastrointestinal hemorrhage	0 (0)	1 (10)	1 (5)	0.591
Hepatorenal syndrome	1 (10)	2 (20)	0 (0)	0.244
Hepatic encephalopathy	0 (0)	2 (20)	1 (5)	0.299

### Virologic response

**Fig. 3:**
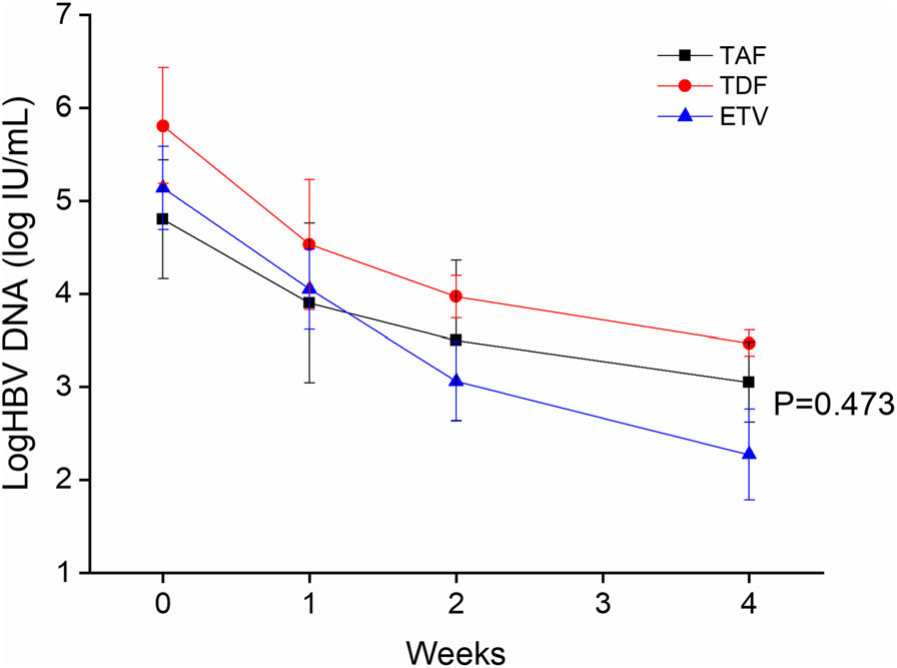
HBV-DNA reduction in serial mean HBV DNA by week 4 in the three groups. ETV, entecavir; TAF, tenofovir alafenamide; TDF, tenofovir disoproxil fumarate

Each group displayed significant reduction of HBV DNA loads in first 4 weeks, which were reduced from baseline 4.80 ± 0.73 to 3.05 ± 0.43 (t = 2.245, *P* = 0.046) in TAF group, from 5.81 ± 0.71 to 3.47 ± 0.33 (t = 3.679, *P* = 0.005) in TDF group, and from 5.13 ± 0.80 to 2.27 ± 0.49 (t = 4.330, *P* = 0.02) in ETV group. Virus load reduction resulted in no statistical significance among three groups, however, ETV group displayed the fastest decline rate (Fig. 3). Additionally, the number of patients with HBV DNA reduction > 2 log at week 4 in the TAF, TDF and the ETV groups was 5 out of 9 (55.5%),3 out of 6 (50.0%) and 10 of 19 (52.6%) (*P* = 0.974). After treatment of 12 weeks, 4 patients in TAF group (66.7%, 4/6) 2 patients in TDF group (50.0%, 2/4) and 6 patients in ETV group (60.0%, 6/10) were virologically undetectable (HBV DNA < 20 IU/mL, *P*>0.05).

### Biochemical response

**Fig. 4:**
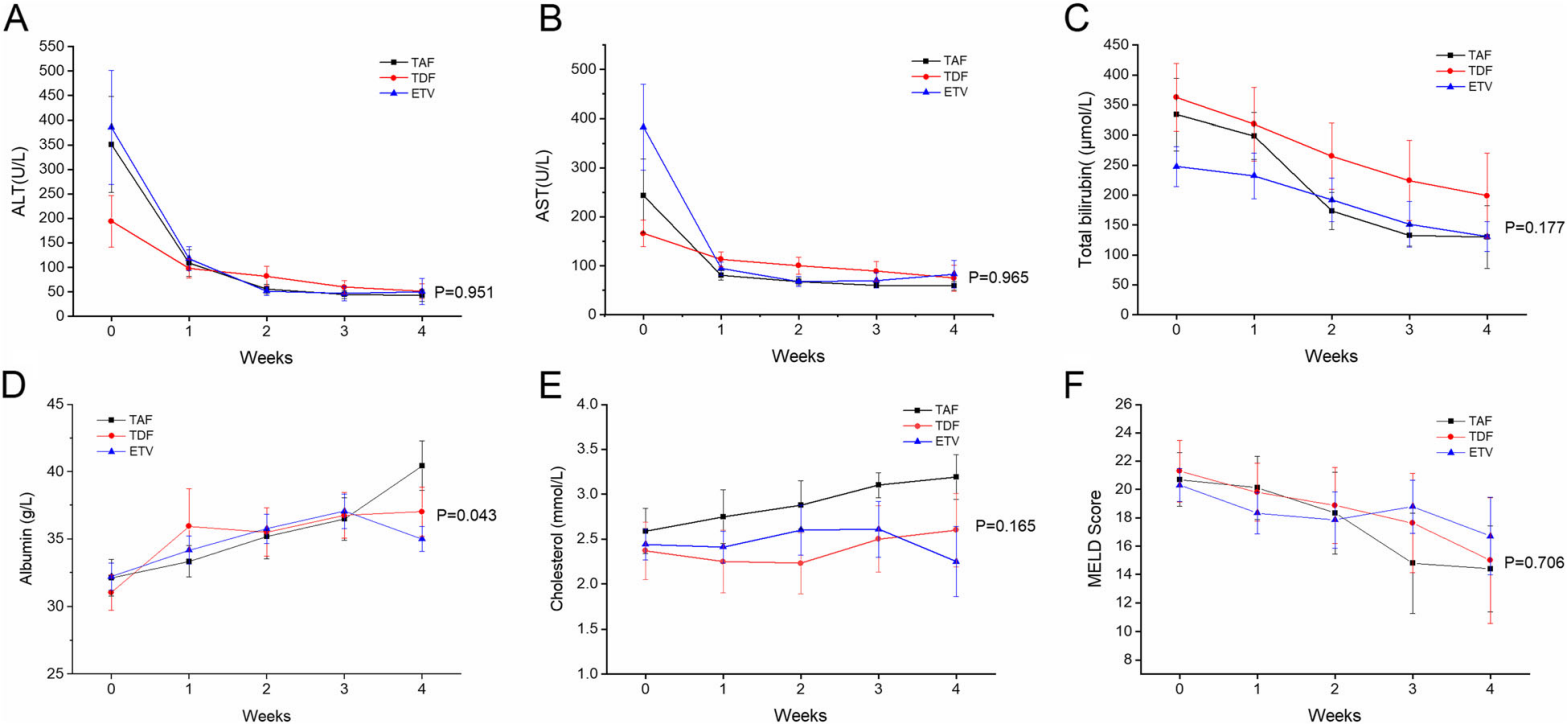
Dynamic changes in serial mean ALT (**A**), AST(**B**), total bilirubin(**C**), albumin(**D**), cholesterol (**E**), and MELD score (**F**) in patients treated with tenofovir alafenamide, tenofovir disoproxil fumarate and entecavir in patients with HBV-related ACLF. ETV, entecavir; TAF, tenofovir alafenamide; TDF, tenofovir disoproxil fumarate; ALT, alanine aminotransferase; AST, aspartate Aminotransferase; MELD, Model for End-stage Liver Disease

As shown in Fig. 4, the decline in serum ALT and AST levels were similar among the TAF, TDF and ETV groups (Fig. 4 A, B). Total bilirubin is the dominant parameter in MELD scoring system and predicting short and long-tern survival of ACLF, we tested bilirubin reduction among the three groups, the median changes of total bilirubin were TAF: -136.75 (IQR -251.23, − 61.65) vs. TDF: -84.10 (IQR -241.15, 55.90) vs. ETV: -36.25 (IQR -77.38, − 5.68) μmol/L, respectively (*P* = 0.057). TAF group showed the fastest decline rate in first two weeks, although there was no significant difference in the serum bilirubin levels at each point among three groups (*P* = 0.177, Fig. 4C). Albumin and cholesterol were exclusively synthesized by hepatocytes. At week 4, the average albumin in the TAF group, TDF group and ETV group were 40.44 ± 1.84, 37.03 ± 1.84, 35.00 ± 0.93 g/L (*P* = 0.043, Fig. 4D), TAF group presented the highest albumin level among three groups. Further analysis showed that patients with TAF treatment showed a significantly higher albumin level than ETV group (TAF vs. TDF, *P* = 0.258; TDF vs. ETV, *P* = 0.293; TAF vs. ETV, *P* = 0.027, respectively). Consistently, TAF group also showed the highest cholesterol level among three groups. After 4 weeks treatment, total cholesterol in the TAF group, TDF group and ETV group were 3.18 ± 0.25, 2.59 ± 0.41, 2.25 ± 0.39 mmol/L, respectively (*P* = 0.165, Fig. 4E). Although there was no statistical difference, the total cholesterol of the TAF group was slightly higher than the other two groups (TAF vs. TDF, *P* = 0.228; TDF vs. ETV, *P* = 0.586; TAF vs. ETV, *P* = 0.071, respectively). It indicated that TAF might be better for the restoration of hepatic synthesis function, but it does not rule out the effect of TAF on total cholesterol, as we knew that TAF does influence the total and LDL-cholesterol levels. Furthermore, the trends in the MELD score of the three groups were also similar (*P* = 0.706, Fig. 4F).

### Safety

There was no drug-related AE reported until follow-up week 48. During the study period, there was no patient who discontinued antiviral therapy due to drug related adverse effects. One patient switched from TAF to ETV at week 6 for the reason of financial burden, others were all well-tolerated, without dosage adjustment or discontinuation.

Additionally, renal dysfunction frequently complicates acute-on-chronic liver failure, up to 50% patients with ACLF experienced acute kidney injury [25]. TDF was reported to cause renal toxicity over long-term use. Thus, we also tested changes in renal function markers in patients with ongoing antiviral treatment. TDF showed unfavorable renal safety even in short term treatment, as shown by increase in serum urea, creatinine, cystatin C and decrease in estimated GFR, although there was no significant difference among the three groups in the change of serum urea (TAF: − 0.41 vs. TDF: 1.46 vs. ETV: 2.73 mmol/L, *P* = 0.635), serum creatinine (TAF: 0.20 vs. TDF: 9.33 vs. ETV: − 2.60 μmol/L, *P* = 0.909), serum cystatin C (TAF: − 0.16 vs. TDF: 0.48 vs. ETV: 0.15 mg/L, *P* = 0.719) and estimated GFR (TAF: 4.35 vs. TDF: − 5.83 vs. ETV: 4.75 mL/min/1.73m^2^, *P* = 0.921) by treatment of week 4. Among the patients who continued follow-up to 48 weeks, no renal-related adverse event, serious adverse renal event, or an event of proximal tubulopathy was observed.

## Discussion

HBV infection is major cause of acute-on-chronic liver failure in Asian countries. Therefore, APASL guideline suggested that nucleos(t) ide analogs should be started immediately in HBV-infected patients [2]. We conducted this prospective cohort study to compare the efficacy and safety of three first-line antiviral agents TAF, TDF and ETV for the treatment of HBV-related ACLF. At the time of preparing the manuscript, this study for the first time to our knowledge assessed the short- and long- term safety and efficacy of the three first line NAs off-label use in patients with ACLF. The results showed that three groups displayed comparable 48-week liver transplant-free survival. Besides, TAF is as effective as TDF and ETV in HBV DNA reduction and liver biochemical responses, and may be more beneficial in synthesis function in the early stages of antiviral therapy. TDF showed a trend of unfavorable renal safety even in short term treatment, although there was no statistical difference in 4-week changes of renal function among three groups. TAF, TDF and ETV are of similar efficacy and safety in short-term and long-term treatment of HBV-ACLF.

Our previous study evaluated the outcome of the three groups of patients receiving ETV, LAM and non nucleos(t) ide analogs controls. The results showed no difference between ETV and LAM groups, thus nucleos(t) ide analogs improved both long-term and short-term outcomes in patients with HBV-related ACLF [26]. Numerous studies demonstrated that ETV had comparable short-term effects with LAM, [14, 27] but more favorable in the long run [28–31]. The first oral nucleoside, LAM, is not recommended by guideline in clinical because of a high incidence of drug resistance. ETV is highly effective for suppressing HBV replication, and significantly lowered rates of resistance, and therefore widely applied in clinical practice.

To date, there are limited studies in efficacy of TDF in HBV-related ACLF. *Wan* et al. [19] demonstrated that TDF was superior to ETV in treating HBV-ACLF in rapidly suppressing the virus at 2 weeks, improving liver function and 48-week survival. While another study from Taiwan [32] compared short-term clinical outcomes of severe acute exacerbations in chronic hepatitis B patients treated with TDF or ETV, suggesting that TDF and ETV generated similar treatment responses and clinical outcomes. Besides, no significant difference in renal safety was observed between these two treatment groups at weeks 1, 2 and 4. Our present study was consistent with the results reported by *Hung* et al. [32] in perspective of TDF and ETV in short-term virologic suppression and biomarkers of both liver and renal function. However, TDF group displayed lower NA-naive rate than other two groups (Table 1), although no significant difference was observed in 48-week transplant-free survival, long-term follow-up is still needed to determine the virologic response of TDF on these patients.

Tenofovir alafenamide is a pro-drug that convert into the pharmacologically active form TFV-DP in the body, as same as TDF. It is primarily excreted in feces, with less than 1% excreted through kidneys. Due to better absorption of this drug into hepatocytes and peripheral blood mononuclear cells, compared with a TDF dose of 300 mg, TAF can reduce the systemic exposure of TFV by more than 90% at a dose of 25 mg or less [33]. Therefore, TAF is considered as a safer alternative to TDF in terms of renal and bone safety, which showed improved safety without compromise in suppressing HBV replication. Several previous studies [21, 22, 34] revealed that TAF had non-inferior antiviral efficacy to TDF, while the indicators of renal function and bone density are more favorable with TAF than TDF during the treatment in chronic hepatitis B (CHB) patients at week 48 and 96. As a result of relatively short period of listing in China, currently there is no study result on the efficacy of TAF in the treatment of HBV-related ACLF in literatures. Consistent with previous reports in CHB, in present cohort study, TAF showed the same effectiveness in virologic and biochemical response by week 4, and similar cumulative rates of 48-week liver transplant-free survival when compared with TDF and ETV. Interestingly, a modest increase in total cholesterol was also observed in TAF group at week 4, although there was no statistical difference compared with TDF and ETV group (Fig. 4E). Similar results have been shown in patients with HIV and chronic HBV when switched TDF to TAF, [35, 36] therefore concurrent evaluation of TAF on lipid metabolism may take into consideration, especially for the aging population with an increasing cardiovascular risk. Besides, patients with TAF treatment presented a significantly higher albumin level than ETV group (Fig. 4D, *P* =0.027). It is known that liver failure is associated with a decrease in serum albumin and cholesterol levels, [37, 38] further studies are required to figure out whether the elevated albumin and cholesterol is a sign of improved hepatocyte synthesis during TAF treatment.

There are however some limitations in our study. First, this is an observational study. Effective randomization can eliminate grouping bias and improve the comparability of research data. However, for a life-threatening disease like ACLF, from an ethical point of view, it is difficult to perform ideal RCT for such severe diseases. Therefore, we conducted a prospective cohort study, and used well-designed matching methods to minimalize patient selection bias. Second, the sample size of the three groups was relatively small after statistical matching. This is partly due to the fact that HBV-related ACLF as a rare severe liver disease is not common these days due to wild use of antivirals. Whereas, our study was a prospective study that for the first time compared the efficacy and safety of TAF, TDF and ETV in HBV-related ACLF, filling the data gap in literatures. Nevertheless, due to the before-mentioned limitations, our results might warrant further larger scale study to evaluate use of TAF in HBV-related liver diseases.

## Conclusions

In conclusion, for the first time we reported that TAF showed the similar efficacy and safety in the short-term and long-term treatment of HBV-ACLF compared with TDF and ETV. Our findings provided new evidence for antiviral treatment options for HBV-related ACLF. Large multicenter prospective studies are required to evaluate the long-term efficacy and safety in HBV-related ACLF population.

## Data Availability

The datasets used during the current study are available from the corresponding author on reasonable request.

## Abbreviations

HBV: Hepatitis B virus
HBeAg: Hepatitis B e antigen
ACLF: Acute-on-chronic liver failure
TAF: Tenofovir alafenamide
TDF: Tenofovir disoproxil fumarate
ETV: Entecavir
HBsAg: Hepatitis B surface antigen
APASL: Asian Pacific Association for the Study of the Liver
MELD: Model for End-stage Liver Disease
INR: International normalized ratio
SD: Standard deviation
ANOVA: One-way analysis of variance
ALT: Alanine aminotransferase
AST: Aspartate Aminotransferase
TBIL: Total bilirubin
WBC: White blood cell count
PLT: Platelet count
eGFR: Estimated glomerular filtration rate
CTP: Child-Turcotte-Pugh
NUC: Nucleos(t) ide analogues
LAM: Lamivudine
CHB: Chronic hepatitis B
